# Entrepreneur-Region Fit and Entrepreneurial Success in China: The Effect of “Confucian” Personality

**DOI:** 10.3389/fpsyg.2021.724939

**Published:** 2021-09-10

**Authors:** Mingjie Zhou, Weiqi Mu, Fugui Li, Yixin Zhou, Duan Huang, Kexin Wang, Jianxin Zhang

**Affiliations:** ^1^CAS Key Laboratory of Mental Health, Institute of Psychology, Chinese Academy of Sciences, Beijing, China; ^2^Department of Psychology, University of Chinese Academy of Sciences, Beijing, China; ^3^School of Journalism and Communication, Nanjing University, Nanjing, China; ^4^Hubei Key Laboratory of Exercise Training and Monitoring, Wuhan Sports University, Wuhan, China; ^5^College of Media and International Culture, Zhejiang University, Hangzhou, China

**Keywords:** entrepreneurship, personality, China, confucianism, interpersonal relatedness, entrepreneur-regional fit

## Abstract

The personality of entrepreneurs is associated with their entrepreneurial success, and the regional personality plays a crucial role in the entrepreneurial ecosystem. Recently, scholars have called for an indigenous personality perspective and combining the personality of entrepreneurs with the regional personality. The current study aimed to investigate the indigenous Confucian personality (e.g., interpersonal relatedness [IR]) and taking an entrepreneur-regional personality fit perspective, allowing testing how entrepreneurs interact with the local ecosystem. Using the personality data of entrepreneurs (*N* = 1,386) from a representative sample across 42 major cities in China, we found that (1) city-level IR is curvilinearly correlated with the annual income of entrepreneurs, with moderate IR associates with the highest income; and (2) the entrepreneur-regional fit analysis further revealed substantial interplay between an entrepreneur and the city. Specifically, entrepreneurs who have moderate IR and run their business in the city also with moderate IR are most likely to have the highest income. This study highlights the usefulness of investigating indigenous personality and the fit perspective in entrepreneurship research.

## Introduction

The Chinese government has encouraged mass entrepreneurship and innovation since 2015. According to the recent government report, there are more than 20,000 newly registered companies every day in 2019 (National Development Reform Commission, 2020), bringing enormous change to the national economy and life of the people. Among those enterprises, only part of them can survive (MyCOS Institute, 2018). Here an important question arises that why do some entrepreneurs succeed while others fail. Personality is found to account for such variation (Zhao et al., 2010). A substantial amount of research, mostly based on the Western countries, has documented the role of personality of entrepreneurs, mostly on Big-Five personality traits, in the entrepreneurial activities and performance (Zhao and Seibert, 2006; Zhao et al., 2010). Despite that, the generalization of the findings on Big-Five to Chinese people is questionable, as the Big-Five construct does not adequately reflect the personality structure of the Chinese people (Cheung et al., 2001).

Indigenous personality research, using an emic method, has identified a unique Chinese personality factor that parallels with the universal Big-Five structure using joint factor analysis (Cheung et al., 2013). Specifically, interpersonal relatedness (IR), also known as the “Confucian” personality, has been found as the core personality of the Chinese people that could distinguish them from the non-Chinese people. Meanwhile, IR is highly relevant in entrepreneurship, as business activities in China are profoundly influenced by the values and norms of Confucianism (Ip, 2009; Chen et al., 2019). Doing business in Confucianism cultures requires well-adaptions to the social mechanism that values “renqing” (reciprocity and relationship orientation), “guanxi” (personalized social network of influence), and “face” (social reputation) (Nolan and Rowley, 2020; Luechapattanaporn and Wongsurawat, 2021). IR, the so-called Confucian personality, taps exactly into these social values and has been found to be a predictive factor to various entrepreneurial activities. For instance, a recent study indicated that Confucian personality is associated with the entrepreneurial vitality in China, whereas the universal Big-Five construct was found to be rather irrelevant (Obschonka et al., 2019). Therefore, Confucian personality is regarded as the leading construct in the current entrepreneurship research due to (1) its centrality in the personality of the Chinese people; and (2) the previously found predictive power of IR in entrepreneurship. However, the research on the association between such indigenous personality and its association with the success of entrepreneurs is scarce.

Entrepreneurs start their business in specific regions. Regional personality, such as the personality of the city (e.g., the population-specific average value on the Big-Five), has been theorized as a regional cultural difference that may allow or restrict entrepreneurship (Obschonka et al., 2013b, 2015). China is an interesting context to investigate entrepreneurship because of its culture-specific social mechanism in doing business (Opper et al., 2017; Liu et al., 2019). Given that IR reflects such indigenous culture, researchers have empirically examined the link between the regional IR (city-level) and the regional entrepreneurial vitality (Obschonka et al., 2019).

Aside from investigating regional characteristics, a new generation of research started to focus on the person-environment fit analysis (i.e., P-E fit). That is, how the characteristic of an individual works together with a region-level characteristic. For instance, Bleidorn et al. (2016) found that people tend to have better self-evaluation if they lived in a city with people who have a similar mind. Regarding entrepreneurship study, one exploratory research recently investigated the link between entrepreneur-city personality fit (e.g., Big-Five) and entrepreneurial success, indicating that the fit research line is insightful and needs to be followed (Zhou et al., 2019).

Given the centrality of IR in Chinese personality and its particular relevance in entrepreneurship, the present study aimed to investigate (1) how IR of entrepreneurs is associated with their entrepreneurial success; (2) how IR of the city is associated with the entrepreneurial success. Moreover, as a response to the burgeoning call for the fit analysis, we further examine how entrepreneur-city IR fit is associated with the entrepreneurial success?

## Literature Review and Hypotheses Development

### Interpersonal Relatedness as the Confucian Personality

Being identified as the sixth personality factor of Chinese personality (aside from the Big-Five), IR was theorized in the Chinese Personality Assessment Inventory (CPAI), reflecting how Chinese people “construct themselves” (Cheung et al., 1996). IR refers to “a strong orientation toward instrumental relationships, emphasis on occupying one's proper place and engaging in appropriate actions; avoidance of internal, external, and interpersonal conflict; and adherence to norms and traditions” (Cheung et al., 2001, p. 425). Particularly, IR reflects the Confucian ideal of realizing humanity in oneself and extending this humanity to others (Cheung et al., 2008). People high in IR tends to value Chinese traditions, care about the interpersonal relationship, avoid face to face conflicts, and give or earn “face” for everyone in the relationship.

### Interpersonal Relatedness and Entrepreneurial Success: From the Entrepreneur Perspective

So far, how IR of entrepreneurs is associated with their entrepreneurial success has barely been empirically investigated. Given that running their own business requires some common qualities with being a hired manager, we may expect a positive link as empirical evidence indicated IR was positively associated with the job performance of a manager. For instance, research using an enterprise manager sample from Hong Kong indicated that IR could positively predict the performance, especially in contexts that require proficiency in dealing with the interpersonal situations, such as working smoothly with people from diverse backgrounds (Kwong and Cheung, 2003). Also, research using manager samples from mainland China also found a positive link between IR and job performance (Gan et al., 2002; Zhang et al., 2012).

As to entrepreneurship, based on the indigenous origin of IR from Confucian philosophy, we can also expect a negative association between IR of entrepreneurs and their entrepreneurial success. As IR reflects the core value of the Confucian philosophy, people high in IR indicated that they are more likely to endorse the Confucian ideology. Confucian philosophy advocates agriculture but restrains commerce. That is, it emphasizes agricultural production and ignores the development of industry and commerce (Hou and Hou, 2002; Herrmann-Pillath, 2015). It is conceivable that if an individual highly endorses Confucian ideology, this person will score high in IR and holds a negative attitude toward the developing commerce and business. Therefore, on the one hand, entrepreneurs with low IR may give prior to business interests (e.g., taking innovation) than to the interpersonal interests, which may increase the chances of success under some circumstances. For instance, IR is found to be negatively associated with innovative behavior, as people high in IR are more sensitive to the risk of losing face or receiving fewer favors from others if they might fail when being innovated (Leung et al., 2014). As innovation is an important quality for entrepreneurial success (Chang et al., 2010), the reduced innovative behaviors due to high IR are likely to impede the entrepreneurial success.

On the other hand, the previous research has reported the positive link between IR and social-related work performance (Kwong and Cheung, 2003). A recent study may also support this rationale that entrepreneurs with higher social-related ability are found to have higher revenue growth due to large social networks, and this effect is especially more prominent in the high relational cultures than in low relational cultures (Batjargal et al., 2019). Given that the Chinese culture is highly relational orientated, we expect the positive association between IR and entrepreneurial success is likely to occur in the sample used in this study.

Therefore, IR might benefit and impede entrepreneurial performance at the same time. A recent study on the Chinese family firms may support both the positive and negative impact of Confucian values in doing business (Luechapattanaporn and Wongsurawat, 2021). On the one hand, the values can cultivate long-term relationships; however, on the other hand, these values may also contribute to the poor management, which would impede the business performance (Luechapattanaporn and Wongsurawat, 2021). Furthermore, Mu et al. (2020) provide more direct evidence on both the negative and positive relationship between IR and the subjective entrepreneurial performance among the young Chinese entrepreneurs. Taken together, we assume that the association between the IR of entrepreneurs and entrepreneurial success would be a non-linear relationship that contains both a positive link and a negative link. We assume that:

H1: The association between IR of entrepreneurs and their entrepreneurial success might be non-linear.

### Interpersonal Relatedness and Entrepreneurial Success: From the Regional Perspective

The behavior and success of entrepreneurs are also influenced by the region where they start and run their business. Based on Lewin's field theory (Lewin, 1939), the behavior of people is affected by the life space. Specifically, research taking *a sociological psychology perspective* focuses on the *regional personality differences*, arguing geographic regions have their own personality (Oishi and Graham, 2010; Rentfrow, 2010). For instance, Rentfrow et al. (2008); Rentfrow (2010) found systematic regional variations (state-level) in the Big-Five personality across the United States. The regional level Big-Five personality is found to influence entrepreneurship in the region (Obschonka et al., 2013b, 2015).

As a response to the call for taking an indigenous personality perspective, researchers investigated the correlates of regional IR and entrepreneurship in China (Obschonka et al., 2019). Specifically, IR was found to be negatively associated with both the manifest entrepreneurship (e.g., rate of newly registered individually owned business) and latent entrepreneurship (e.g., number of entrepreneurship-related search queries in the internet search engine). Although this research focuses on the *regional-level* entrepreneurship vitality, we can also expect a similar negative link between the regional IR and the success of an *individual-level* entrepreneur. According to Rentfrow et al. (2008), people respond, adapt to, or get socialized in line with the regional norms and attitudes. In this sense, an individual entrepreneur will perceive the personality climate in this region and adjust to their behaviors. If the region is high in IR, it means a large proportion of people in this city tend to score high in IR and advocate the traditional Confucian value, thus stressing agriculture while limiting the development of commerce, industry, and business. The high regional IR also reflects the macro culture that emphasis on the social harmony and conformism instead of encouraging the prototypical qualities of entrepreneurs, such as rule-breaking or risk-taking (Zhang and Arvey, 2009; Obschonka et al., 2013a). The high regional IR may discourage entrepreneurs from breaking the rules or taking innovative behaviors because entrepreneurs can sense what is favored and what is not. When perceiving something is not encouraged in the environment, people tend to suppress themselves from doing such things (Matthes et al., 2018). Given innovation is an essential factor for the entrepreneurial success, we believe that compared to a region with extremely high IR, a relatively low IR region will develop a more favorable entrepreneurial culture that could nurture entrepreneurship and facilitate success.

On the other hand, we may also expect a positive link between the regional IR and the success of entrepreneurs because how regional IR associates with the entrepreneurial vitality might be different from how regional IR associates with entrepreneurial success. In particular, the regional IR may negatively correlate with the enthusiasm of starting up a business in this region because Confucianism does not value business and looks down on merchants (Herrmann-Pillath, 2015). However, the regional IR may positively correlate with the entrepreneurial performance because Confucianism values the establishment and maintenance of the instrumental relationship (e.g., with strangers aim at obtaining specific resources), thus creating a friendly social climate for entrepreneurs to run business (Hwang, 2010), which in turn, may promote success.

Taken together, it could be that from extremely low to moderate regional IR, the regional IR is positively associated with the entrepreneurial success because the regional climate might be more agreeable for entrepreneurs to navigate business with interpersonal resources. However, from moderate to extremely high regional IR, the regional IR is negatively associated with the entrepreneurial success because the regional climate might become discouraging for taking innovation. Therefore, we assume that:

H2: The association between regional IR and entrepreneurial success assembles as an inverted U-curve.

### Interpersonal Relatedness and Entrepreneurial Success: From an Entrepreneur-Region Fit Perspective

We have discussed how IR of entrepreneurs and regional IR may respectively associate with the entrepreneurial success. The question remains how IR of entrepreneurs and regional IR jointly affect the entrepreneurial success. The P-E fit perspective may help to probe this question. The P-E fit (similarity, match, or congruence) perspective indicates that if the characteristics of a person go well with the environmental characteristics, this person will be more likely to achieve success (Zhou et al., 2019).

What is an ideal entrepreneur-region IR fit for promoting success? Evidence showed that positive self-evaluation predicts the performance (Judge and Bono, 2001) and being with like-minded others facilitates the positive self-evaluation. As mentioned earlier, Bleidorn et al. (2016) found that people had positive evaluations about themselves if they lived in a city with a similar personality. Therefore, we may expect that if entrepreneurs with the certain IR level runs the business in a city with similar IR levels, an entrepreneur will be more likely to succeed due to the enhancing effect of positive self-evaluation. However, an entrepreneur-region IR fit can be complex and lacks adequate research to formulate the hypotheses; we now ask an open question,

Q1: What IR fit is beneficial for entrepreneurial success?

## Method

### Participants

In this study, a representative Chinese urban resident sample was used from the Project of Factors on Mental Health Survey conducted by the Institute of Psychology, Chinese Academy of Sciences (H20020). This sample was collected from 42 major cities of China across six different regions with a quota sampling method. People reported their personality and demographic information on a paper-pencil questionnaire. Before administrating the survey, researchers orally informed all the participants of the purpose, benefits, and confidential policy of the study. All the participants were informed that they could withdraw from the study at any time. The total sample included 26,405 urban residents of China, ranging from 104 to 2,862 respondents from each city (*M* = 548 ± 683 per city). The participants aged from 18 to 65 years (*M* = 33.65 ± 9.75), with 56.3% were female.

Among all the participants, 1,386 were entrepreneurs. It has to be noted that the definition of an entrepreneur is different from that of in the Western culture. Based on the prior Chinese research and the Chinese national occupational category (Zhou et al., 2019), we defined an entrepreneur as (1) the private business owners and (2) self-employed entrepreneurs. Specially, this study focused on an individual small business which belongs to a natural person or household. We have 548 private business owners and 838 self-employed entrepreneurs.

### Measures

#### Interpersonal Relatedness of Entrepreneurs

Interpersonal relatedness was measured with the subscale from the Cross-cultural Personality Assessment Inventory (CPAI-2) used in the study by Obschonka et al. (2019) (as shown in the Appendix for the scale). Entrepreneurs rated their agreements on each item (1 = strongly disagree to 5 = strongly agree). A mean score was created as the indicator for IR. The Cronbach's α is 0.84.

#### Regional IR

All the urban residents (*n* = 26,405) indicated their agreement on the IR items. An average score was created for each city according to the IR scores of residents. The regional IR was thus achieved on a city level.

#### Entrepreneurial Success

Financial indicators are typical measurements for the entrepreneurial success. In this study, the entrepreneur participants are small business owners. Small business entities are characterized as the primary source of income, which bounds closely with the family needs (Carland et al., 1984). Besides, following previous entrepreneurial research in China, financial indicators of small business owners usually reflect how much they can provide for their families (Zhou et al., 2019). Therefore, we used the annual family income of the owners as the indicator of entrepreneurial success by asking “What is the total annual income of your family at present?”

#### Control Variables

In the study, both the entrepreneurs-related and regional-related control variables were included. The entrepreneurs-related variables are gender, age, and education year as the prior research found these variables relevant for the entrepreneurial success (Zhou et al., 2019). The regional control variable is the city-level gross domestic product (GDP) per capita, as regional economic competitiveness is a predictive factor for the entrepreneurial activities (Audretsch et al., 2012). In addition, we controlled for the sample size of cities to make it comparable among the cities with substantial sample differences (Chen et al., 2007).

### Statistical Analysis

Although simple difference scores were frequently used to assess fit (e.g., absolute difference score), which was identified with major methodological problems, such as oversimplifying different fit situations or covering up the independent effect of each specific predictor (Edwards, 1993). Researchers recommended polynomial regression because it can overcome these major problems (Edwards, 2002). Specifically, it included the examination of two independent predictors (i.e., IR of entrepreneurs and regional IR), the combination of these two predictors (i.e., the product of IR of entrepreneurs and regional IR), and higher-order terms (i.e., squares of IR of entrepreneurs and regional IR) in the fit analysis. The five terms allowed fine-grained interpretations for the joint effects of two predictors.

The analyzing steps were as followed. First, we made some transformations to the original values of the independent variable (IV) and dependent variable (DV). About the IV, we centralized both the IR of entrepreneurs and regional IR to avoid multicollinearity before creating higher-order terms. About the DV, we followed prior research by using the logged value of annual family income, as this transformation normalizes the original values, which allows for polynomial regression (Zhou et al., 2019).

Second, we conducted a multilevel hierarchical regression. In the first block, the age, gender, education year, city-level GDP per capita, and city sample size were included as controlled variables. In the second block, the IR of entrepreneurs and regional IR were included to examine their independent linear effects on entrepreneurial success. In the third block, the three higher-order terms (product of IR of entrepreneurs and regional IR, square of IR of entrepreneurs, and square of regional IR) were included to examine the curvilinear effect, as well as the entrepreneur-region joint effect. The equation for polynomial regression is as follows.


$$\begin{array}{l} Enterpreneurial\;success=b_{0}\\ +b_{1}\times enterpreneur's\;IR+b_{2}\times regional\;IR\\ +b_{3}\times enterpreneur's\;IR\;squred+b_{4}\times enterpreneur's\;IR\\ \times regional\;IR+b_{5}\times regional\;IR\;squred+e. \end{array}$$

Third, additional tests were conducted to examine the different situations of entrepreneur-region fit (congruence/incongruence). Recently, researchers provided an R package with instructions to inspect the fit effect (as shown in details in Humberg et al., 2019). Then a 3D response surface was plotted to facilitate the interpretation of the results. For clarity, the testing steps and interpretations are demonstrated in the results section.

## Results and Discussion

The descriptive statistics and correlation analysis results are shown in Table 1.

**Table 1:** The descriptive statistics and correlations for the two samples.

**Research Variables**	***M (SD)***		**1**	**2**	**3**	**4**	**5**	**6**	**7**	**8**
	**self-employed entrepreneurs**	**Private business owners**								
1. Age	33.50 (11.95)	33.21 (11.15)	–	−0.06	−0.00	0.08^*^	−0.01	0.01	0.03	−0.06
2. Gender	1.48 (0.50)	1.36 (0.48)	−0.04	–	−0.02	−0.11^**^	−0.05	0.04	0.11^**^	−0.09^*^
3. Education	3.39 (1.11)	4.13 (1.14)	−0.03	−0.06	–	−0.16^**^	−0.01	−0.08^*^	0.09^*^	0.33^***^
4. City Sample Size	1096.27 (1052.73)	1054.90 (1028.25)	0.07	−0.00	−0.04	–	0.29^***^	−0.07^*^	−0.05	−0.10^**^
5. GDP per Capita	53324.39 (20070.80)	56825.66 (19579.19)	−0.05	−0.01	0.10^*^	0.22^***^	–	0.03	0.07^*^	0.15^***^
6. E-IR	3.27 (0.47)	3.19 (0.50)	0.03	0.08	0.00	−0.07	0.03	–	0.16^***^	−0.06
7. R-IR	3.24 (0.08)	3.24 (0.07)	−0.07	−0.01	0.22^***^	0.03	−0.00	0.16^***^	–	−0.07
8.Entrepreneurial success	4.67 (0.42)	4.95 (0.48)	−0.10^*^	−0.08	0.23^***^	−0.05	0.16^***^	−0.01	−0.03	–

*E-IR, entrepreneur's IR; R-IR, regional IR. Correlations for self-employed entrepreneurs (n = 838) are presented above the diagonal, and correlations for private business owners (n = 548) are presented below the diagonal*.

* *p < 0.05*;

** *p < 0.01*;

*** *p < 0.001*.

### Interpersonal Relatedness of Entrepreneurs and the Entrepreneurial Success (H1)

Our first hypothesis proposed a non-linear association between IR of entrepreneurs and the entrepreneurial success. As shown in Table 2, this hypothesis was supported among private entrepreneurs (*b*_3private entrepreneurs_ = −0.02, *SE* = 0.01, *p* < 0.05), but not among the self-employed entrepreneurs (*b*_3self−employed entrepreneurs_ = −0.01, *SE* = 0.01, *p* >0.05). The coefficients of quadratic terms (IR of entrepreneurs) only reached a significant value in one sample. Thus, H1 is partially supported. The inverted U-curve meant that, from the extremely low to medium levels of IR of entrepreneurs, annual income of the private entrepreneurs increased with their levels of IR; however, from the medium to high levels of IR of entrepreneurs, the annual income of the private entrepreneurs decreased with their levels of IR.

**Table 2:** Results of the polynomial regressions of entrepreneurial success on the IR of entrepreneurs and regional IR.

	**Self-employed entrepreneurs (** ***n*** **=** **838)**			**Private business owners (** ***n*** **=548)**		
	***B***	***se***	***95% CI***	***B***	***se***	***95% CI***
Constant (*b*_0_)	4.48^***^	0.07	4.335, 4.626	4.87^***^	0.12	4.624, 5.109
E-IR (*b*_1_)	−0.02	0.01	−0.043, 0.011	−0.01	0.02	−0.044, 0.034
R-IR (*b*_2_)	−0.04^**^	0.01	−0.069, −0.017	−0.05^*^	0.02	−0.098, −0.011
E-IR ^2^ (*b*_3_)	−0.01	0.01	−0.024, 0.006	−0.02^*^	0.01	−0.039, 0.005
E-IR × R-IR (*b*_4_)	0.01	0.01	−0.016,0.031	0.00	0.02	−0.038,0.045
R-IR ^2^ (*b*_5_)	−0.04^***^	0.01	−0.052,−0.020	−0.04^**^	0.02	−0.075, −0.012
Age	0.00	0.00	−0.004, 0.001	−0.00^**^	0.00	−0.008, −0.001
Gender	−0.06^*^	0.03	−0.112, −0.007	−0.06	0.04	−0.137, 0.023
Education	0.11^***^	0.01	0.085, 0.132	0.09^***^	0.02	0.052, 0.127
City Sample Size	−0.06^***^	0.01	−0.090, −0.035	−0.03	0.02	−0.071, 0.007
GDP per Capita	0.09^***^	0.01	0.059,0.114	0.07^***^	0.02	0.029, 0.100
R^2^ for overall model	0.18			0.11		
ΔR^2^ above baseline model with control variable	0.03			0.03		
Congruence (x = y) line						
Slope (*a*_1_)	−0.06^**^	0.02	−0.094, −0.024	−0.06^*^	0.03	−0.113, −0.006
Curvature (*a*_2_)	−0.04^*^	0.02	−0.066, −0.009	−0.06^**^	0.02	−0.108, −0.017
Incongruence (x = -y) line						
Slope (*a*_3_)	0.03	0.02	−0.013, 0.067	0.05	0.03	−0.014, 0.113
Curvature (*a*_4_)	−0.05^**^	0.02	−0.088, −0.017	−0.07^*^	0.03	−0.131, −0.006

*E-IR, entrepreneur's IR; R-IR, regional IR*.

* *p < 0.05*;

** *p < 0.01*;

*** *p < 0.001*.

### Regional IR and Entrepreneurial Success (H2)

Our second hypothesis proposed an inverted U-shaped association between regional IR and entrepreneurial success. As shown in Table 2, this hypothesis was fully supported among both the private entrepreneurs (*b*_5private entrepreneurs_ = −0.04, *SE* = 0.02, *p* < 0.01) and self-employed entrepreneurs (*b*_5self−employed entrepreneurs_ = −0.04, *SE* = 0.01, *p* < 0.001). The inverted U-curve meant that, from the extremely low to medium regional IR, annual income of entrepreneurs increased with regional IR levels; however, from medium to high regional IR, the annual income of entrepreneurs decreased with regional IR levels. Thus, H2 was fully supported.

### Entrepreneur-Regional IR Fit and Entrepreneurial Success

To answer the third question about the fit effect of IR of entrepreneurs and regional IR, we calculated the slopes and curvatures along the (in)congruence lines. As shown at the bottom of Table 2, the response surface analyses revealed two identical arched surfaces for both the samples, indicating consistent fit effects among both the private entrepreneurs and self-employed entrepreneurs. Overall, the fit effect suggested that if entrepreneurs with moderate IR ran their business in a city where people were alike with moderate IR, they would be the most likely to have the highest annual income. Specifically, the fit analyses yielded two meaningful results supporting this overall claim.

First, if the entrepreneur and city were alike in IR at low-levels or high-levels, the annual income would be lower than that in the moderate levels. This was because the curvature along the congruence line reached significance in both the samples (*a*_2private entrepreneurs_ = −0.06, *SE* = 0.02, *p* < 0.01, *a*_2self−employed entrepreneurs_ = −0.04, *SE* = 0.02, *p* < 0.05) and the negative coefficients indicated inverted U-curves. As noted above, approximately, the moderate fit was associated with the highest annual income along the inverted U-curve. Second, if entrepreneurs ran their business in a city where people were alike in IR, they would earn more money when IR was low compared with when IR was high. This was because the slopes along the congruence line reached significance with negative coefficients in both the samples (*a*_1__private entrepreneurs_ = −0.06, *SE* = 0.03, *p* < 0.05, *a*_1__self−employed entrepreneurs_ = −0.06, *SE* = 0.02, *p* < 0.01), suggesting that higher fits were associated with the lower annual income. Besides, if the entrepreneurs and the city were different in IR, then the larger the difference, the lower was the annual income. This was because the curvature along the incongruence line reached significance in both the samples (*a*_4__private entrepreneurs_ = −0.07, *SE* = 0.03, *p* < 0.05, *a*_4__self−employed entrepreneurs_ = −0.05, *SE* = 0.02, *p* < 0.01) and the negative coefficients indicated inverted U-curves.

**Figure 1:**
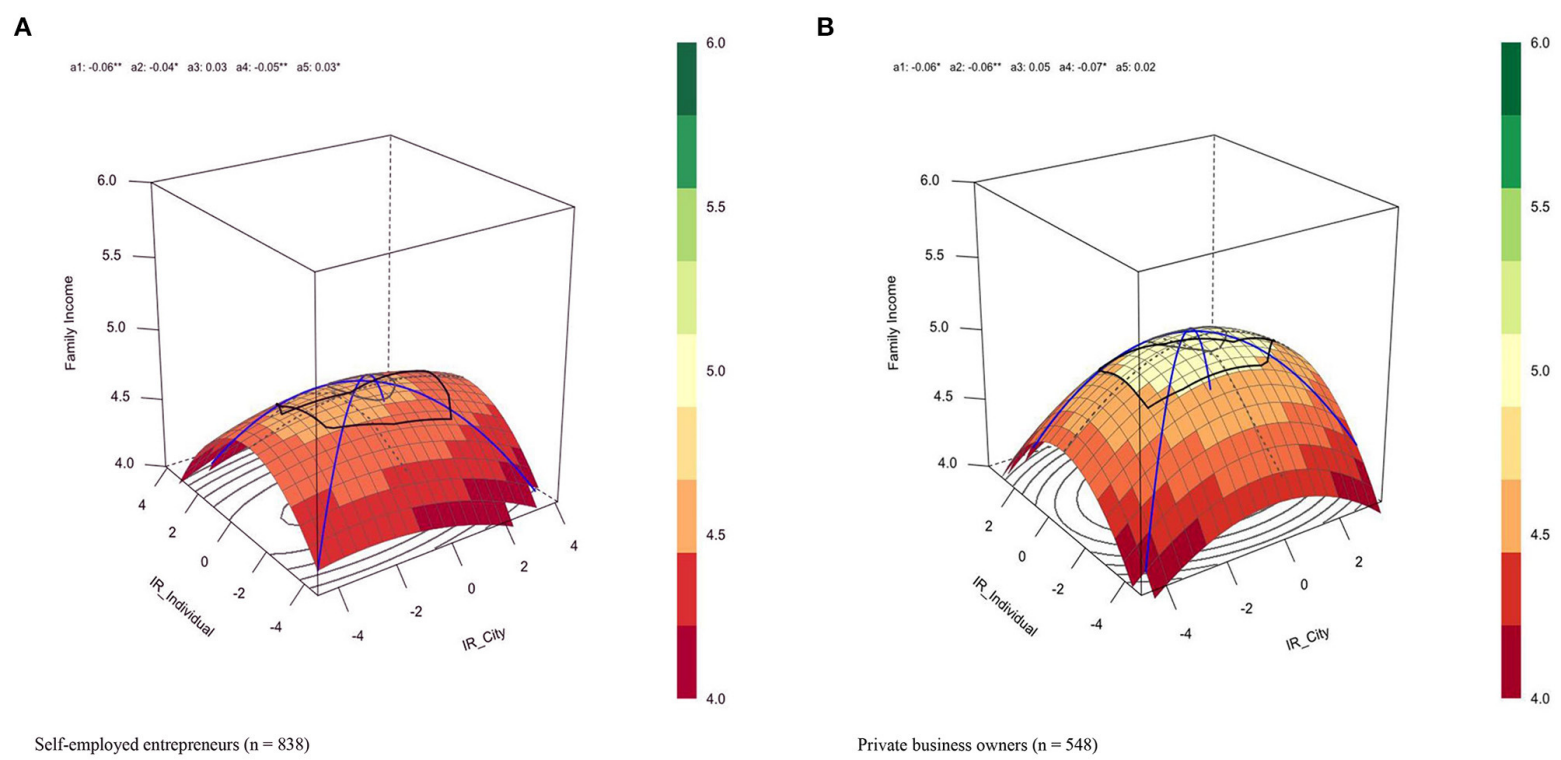
Response surface plots for **(A)** self-employed entrepreneurs and **(B)** private business owners. E-IR, entrepreneur's IR; R-IR, regional IR.

As depicted in the 3D surface in Figure 1, the vertical axis shows the amount of annual income, with different colors representing different income levels. The right corner of the surface represented the fit that the entrepreneurs scored extremely low in IR, whereas the city scored extremely high in IR. By contrast, the left corner of the surface represented the fit that the entrepreneurs scored extremely high in IR, whereas the city scored extremely low in IR. From these two corners to the middle part of the surface, the differences between the IR of the entrepreneurs and regional IR became smaller because both values were approaching the moderate levels, along with this, the annual income was higher. Thus, we concluded that the larger the difference in IR, the lower the likelihood to succeed.

### Discussion

Using a representative Chinese small business entrepreneur sample, we investigated how the IR of entrepreneurs, the city-level regional IR, and the joint effect of IR of the entrepreneurs and regional IR on entrepreneurial success. We found that the IR of the entrepreneurs was curvilinearly associated with their annual income among the private entrepreneurs but not among the self-employed entrepreneurs, which partially supported our hypothesis (H1). Besides, the city-level regional IR was curvilinearly associated with the annual income of the entrepreneurs in both samples, with moderate IR associated with the highest annual income, which fully supported hypothesis (H2) of this study. The fit analyses revealed that entrepreneurs with moderate IR run their business in a city also with moderated IR were most likely to have the highest annual income.

This study research is the first empirical research that explicitly investigated the association between IR of the entrepreneurs and their entrepreneurial success from the entrepreneur-region fit perspective. Given its explanatory role in the behavior of the Chinese people and the particular relevance regarding doing business, we provide empirical evidence about the relationship of Confucian trait with their personal success. That is, being moderate in IR seems a favorable personal characteristic for a successful business among the private entrepreneurs in China, while not self-employed entrepreneurs. The different results in the two samples may be due to the differences regarding *scale* and *operation mode* between the self-employed enterprises and the private enterprises. The self-employed enterprises are mostly run within family numbers and have a relatively smaller scale, whereas private enterprises were run with employees and have a relatively larger scale. Therefore, the lower IR in the self-employed enterprises can reduce the harm of cronyism to the performance of the enterprise. While for the private enterprise owners, extremely low IR might bring the employees a harsh and inhospitable feeling. The future research may consider the composed structure of employees as a covariate when investing IR of the entrepreneurs and their success. In addition, this finding should be interpreted with a recent finding which reported that, compared with moderate IR, lower IR and higher IR are associated with the higher levels of perceived entrepreneurial performance (Mu et al., 2020). The inconsistent findings on the link between IR and the entrepreneurial performance might be because of the association between IR and subjective evaluation of entrepreneurial success is different from that of objective indicators of the entrepreneurial success. Future research is called for to validate both the findings and examine the potential difference.

The city-level regional IR was curvilinearly associated with the annual income of the entrepreneurs as expected, suggesting that a city with moderate IR may provide the most fertile environment for profitable small business enterprises. This finding speaks to previous research, which points out that the perceptions, attitudes, and behaviors of a certain population together constitute a latent “entrepreneurial spirit” in the region, which may translate into the regional entrepreneurial activities (Audretsch and Fritsch, 1994; Sternberg, 2009). By examining the city-level IR, we specify such “entrepreneurial spirit” in China as moderate IR, which can create a favorable atmosphere for promoting the success of the small businesses. Specifically, the region with moderate IR tends to facilitate the successful business because people in this region basically merit in maintaining harmonious reciprocal relations. In such a relational culture, entrepreneurs are more likely to forge satisfactory social networks and smooth communication. Nevertheless, the promoting effect of moderate regional IR on entrepreneurial success seemed different for entrepreneurs with different IR levels.

The fit analyses revealed that this promoting effect was most prominent for the entrepreneurs who also have moderate IR. This finding reflected a *similarity attraction* effect, suggesting shared tendency in IR between the entrepreneurs and the population of their city paves ways for a successful business. By contrast, if the entrepreneurs with moderate IR run a business in a city with either extremely low IR or extremely high IR, their income will be remarkably lower than those with moderate IR. Therefore, we believe that the potential of entrepreneurs with moderate IR in running a successful enterprise, such as socially adequate and intellectually innovative, can get fully exerted when the population in the same city is also like-minded.

## Implication, Limitation, and Future Direction

### Theoretical Implications

This research contributes to the literature in four aspects. First, we enrich the personality entrepreneurship research by introducing the Confucian trait and provide robust empirical evidence for its usefulness by using a representative Chinese sample. Second, entrepreneurship is regarded as “regional events” (Feldman, 2001) and regional personality is a crucial ingredient in such events (Obschonka et al., 2015), which points to the necessity of investigating (regional) personality. To the best of our knowledge, only one research has explicitly examined IR and entrepreneurship from a macro perspective by focusing on the regional entrepreneurial vitality (Obschonka et al., 2019). Therefore, investigating IR across the major cities increases the knowledge of how IR shapes entrepreneurship in China. Third, researchers regard entrepreneurship as interactions, meaning that the entrepreneurs interact with society economically and culturally (Hisrich et al., 2007). The fit analysis prevails in the entrepreneurship research because it centralizes the interactions and puts a dual emphasis on the people and the environment (Edwards et al., 1998). Therefore, our research contributes by taking the *fit* perspective, providing a fine-grained picture of how the indigenous personality of the entrepreneurs and the city as macro-level culture jointly determined the success of the entrepreneurs. Last but most important, this research points out an ideal matching on Confucian personality between entrepreneur and the city, speaking to the seemingly “*paradox of personal gains and social lost*” found in the previous studies on IR and entrepreneurship. Specially, Mu et al. (2020) argue that IR of entrepreneurs, on the individual level, is positively related to entrepreneurial performance, suggesting *a person gain effect* on IR. However, Obschonka et al. (2019) indicate that the IR of the city, on a regional level, is negatively related to the regional entrepreneurial vitality, suggesting *a social lost effect* in IR. This research, taking an integrated fit perspective, articulates that a person-city balance should be kept at a moderate IR level to achieve the entrepreneurial success.

### Practical Implications

This research has the following practical implications. First, entrepreneurs should increase their knowledge about the social mechanism of doing business in China and the adaptivity of indigenous personality in such mechanism. Particularly, moderate IR may enable private entrepreneurs to expand the instrumental relationship, nurture the long-term relationship, and keep motivated to innovate. The private entrepreneurs may mindfully cultivate and conduct themselves accordingly. Second, when considering where to start their business, entrepreneurs may take into account whether the IR of the city agrees with their own IR. Selecting the city based on IR fitness, entrepreneurs may be more likely to succeed because they tend to feel well-adapted or enabled in the city. In contrast, mismatching in IR may lead to the inadaptation for the entrepreneurs. For instance, if entrepreneurs with high IR run their business in a city with low IR, they might feel a sense of “fish in the shark pond” that they highly value interpersonal harmony and cooperation, whereas the city ecosystems are highly competitive (Obschonka et al., 2019). Third, policymakers may consider cultivating a moderate IR culture, which does not excessively encourage rule-breaking or self-reliance that Western culture may value for the entrepreneurship (Zhang and Arvey, 2009). In Chinese society, IR-related values, such as interpersonal harmony and reciprocal dependence, are also essential for running a successful business. One recent research may also support this claim that IR was found to have two folds, with one stressing *relationship orientation* values, for instance, appreciating harmony, the other focusing on *conduct oneself* values, for instance, abiding by discipline (Zhou et al., 2021). Low regional IR, in this sense, may not only exert a negative influence on the relational atmosphere but also undermine the rule consciousness, which is the cornerstone of the modern market economy. Policymakers thus need to advocate cooperation and seeking mutual benefits for the entrepreneurship from a regional macro-level.

### Limitation and Future Direction

The findings of this research have to be interpreted with the following limitations. First, as an exploratory study, we found a *fit effect* between city-level IR and the family income of entrepreneurs. Future research may consider using more precise and objective regional economic indicators, such as the city-level tax revenue contributed by these enterprises. Also, entrepreneurial success should not be evaluated only by financial indicators. Future research may include multiple indicators, such as satisfaction of the entrepreneurs (Dej, 2010), to quantify entrepreneurial success. Second, we have included the self-employed and private-owned enterprises for sample diversity. However, those enterprises may belong to various economic sectors. The previous research has underscored the heterogeneity of enterprises that should be considered when investigating entrepreneurship (Davidsson, 2016). Future research may need to control for such heterogeneity or compare among enterprises based on their attributions. Third, despite that IR fitness finding is insightful, we still do not know how different fits translate into the success of the entrepreneurs. A promising next step might be investigating the self-efficacy of entrepreneurs, as previous research has hinted at its crucial role in predicting performance (Newman et al., 2019).

## Conclusion

The present research found that cities with moderate IR may provide the “entrepreneurship-friendly” context for entrepreneurs to yield successful business and that the entrepreneurs who have moderate IR are most likely to succeed. These findings contribute by stressing the importance of investigating indigenous “Confucian” personality in the entrepreneurship research from an entrepreneur-region fit perspective, and explicitly pointing out that an ideal fit for IR is remaining moderate on both parts. Therefore, entrepreneurs should practice accordingly by balancing the interpersonal harmony with the willingness to take potential personal risks for the innovation instead of emphasizing one side at the expense of another. In addition, they may need to consider the fitness of IR between themselves and the city when starting their business. Regional policymakers should acknowledge the traditions of values and norms that date back to Confucianism and uphold the motivation and agency of the local population to promote entrepreneurship at the same time.

## Data Availability Statement

The raw data supporting the conclusions of this article will be made available by the authors, without undue reservation.

## Ethics Statement

The studies involving human participants were reviewed and approved by Ethical Review Board of the Institute of Psychology, Chinese Academy of Sciences. Written informed consent for participation was not required for this study in accordance with the national legislation and the institutional requirements.

## Author Contributions

JZ and MZ developed the research project. DH and MZ carried out the data collection. YZ and WM carried out the data analysis. KW wrote the first draft. WM, FL and MZ revised the manuscript. All authors contributed to the article and approved the submitted version.

## Funding

This work was supported by the National Natural Science Foundation of China (Grant No. 71774156).

## Conflict of Interest

The authors declare that the research was conducted in the absence of any commercial or financial relationships that could be construed as a potential conflict of interest.

## Publisher's Note

All claims expressed in this article are solely those of the authors and do not necessarily represent those of their affiliated organizations, or those of the publisher, the editors and the reviewers. Any product that may be evaluated in this article, or claim that may be made by its manufacturer, is not guaranteed or endorsed by the publisher.

## Appendix

Following are items measuring the interpersonal relatedness (IR) of entrepreneurs which was used in the study by Obschonka et al. (2019).

1. Inviting someone out to dinner has to be done in style in order to keep up appearances.
2. I am usually very particular about the way I dress because I do not want others to look down on me.
3. I would rather cut down on my regular expenses, but when it comes to inviting people out or giving presents, I must be generous.
4. Even if I do not have much money, I would still try to buy a presentable coat.
5. To avoid mistakes in life, the best thing to do is to listen to the elders' suggestions.
6. Students should concentrate on their studies and not get distracted by what is happening in the society.
7. A woman's chastity is more important than her life.
8. I try my best to listen to my parents out of filial piety.
9. In order to avoid offending others, it is best not to show off too much.
10. When dealing with organizations, things can work out more smoothly through the connections of friends working inside.
11. When a friend borrows something from me and does not return it, I often feel uneasy about asking him/her to give it back.
12. Blood is thicker than water, and no matter what, one's feelings for one's family are always stronger than for outsiders.
13. One can avoid making serious mistakes by always following tradition.
14. Rules and laws should be strictly enforced and should be without exception.
15. I believe traditional ideas or concepts should not be torn down.
16. It would be great if everyone had a similar way of thinking or a similar value system.
17. I try my best to maintain harmony in my family because I believe that if a family lives in harmony, all things will prosper.
18. When facing a dilemma, I can always arrive at a compromise.
19. It is a virtue to tolerate everything.
20. I follow the saying that “Those who are contented are always happy” as a principle in life.

Entrepreneurial success: “What is the total annual income of your family at present?”
